# Editorial: The influence of environmental conditions on chloroplast functioning and development

**DOI:** 10.3389/fpls.2024.1517094

**Published:** 2024-11-25

**Authors:** Małgorzata Adamiec, Małgorzata Pietrowska-Borek, Robert Luciński

**Affiliations:** ^1^ Department of Plant Physiology, Faculty of Biology, Institute of Experimental Biology, Adam Mickiewicz University, Poznań, Poland; ^2^ Department of Biochemistry and Biotechnology, Faculty of Agronomy, Horticulture and Biotechnology, Poznań University of Live Sciences, Poznań, Poland

**Keywords:** light stress, osmotic stress, water stress, temperature stress, nutrient availability, chloroplast, chloroplast development

Chloroplasts are essential organelles in plant cells, primarily responsible for photosynthesis, fatty acid synthesis, amino acid production, hormone biosynthesis, and nitrogen and sulfur assimilation (Mahapatra et al., 2024). The starting point for chloroplasts development are proplastids, which also have a vital role in plant embryo development (Liu et al.). Differentiating proplastids into chloroplast is a complex process that requires, among others, the import of numerous proteins encoded by the nuclear genome and the synthesis and assembly of thylakoid membranes. Environmental stresses, such as light, temperature, water, nutrients, and CO_2_ levels, can significantly impact chloroplast development and functioning. Understanding how these factors influence chloroplast differentiation and the effectiveness of their performance is crucial for improving plant health and productivity, especially in changing environmental conditions (Mahapatra et al., 2024).

This Research Topic brings together a collection of studies that delves into mechanisms of proplastids development, the various environmental factors that affect chloroplasts, the underlying mechanisms involved, and the remarkable adaptations chloroplasts have evolved to cope with these conditions.

## Light intensity and quality

The intensity and spectral quality of light are crucial determinants of chloroplast performance.

The quality and intensity of light affect both the structural elements of the photosynthetic machinery, such as the composition and arrangement of thylakoid complexes, as well as the photosynthetic electron transport. This is reflected in the ATP/NADPH balance, which is one of the main factors regulating the efficiency of CO_2_ assimilation processes (Zhang et al., 2024). High light intensity can enhance photosynthetic activity but may also lead to photoinhibition, impairing photosynthetic electron transport and primarily affecting photosystem II (PSII). Plants mitigate this damage through different mechanisms, such as the dissipation excess light energy as heat. Conversely, low light conditions can limit chloroplast development and reduce photosynthetic efficiency. The spectral quality of light, particularly the wavelengths absorbed by chlorophyll (mainly blue and red light), also significantly influences photosynthesis (Paradiso and Proietti, 2022).

## Temperature

Temperature is a critical factor influencing chloroplast function. High temperatures can cause the denaturation of photosynthetic enzymes and disrupt membrane integrity, while low temperatures can slow down metabolic processes and reduce enzyme activity (Schwenkert et al., 2022). Chloroplasts adjust thylakoid membrane composition to maintain fluidity during temperature stress. The increased content of unsaturated fatty acids at low temperatures prevents rigidity, while at high temperatures, they increase saturated fatty acids to prevent excess fluidity (Schwenkert et al., 2022). These adjustments help maintain the integrity and functionality of the photosynthetic apparatus under varying temperature conditions.

## Water availability

Water is a fundamental component of all life processes. It also serves as an electron donor in the photosynthesis light-dependent reactions of photosynthesis. Under drought stress, plants undergo stomatal closure to preserve water, which limits the diffusion of CO_2_ into the leaf mesophyll and subsequently restricts the rate of photosynthesis. In response to water deficit, plants synthesize and accumulate abscisic acid (ABA), which signals stomatal closure (Razi and Muneer, 2021). The recovery of chloroplasts from drought stress depends on the leaves’ age. Mature leaves recover better than young or old leaves, reflected in the increased chloroplast surface area (Jahan et al.).

## Salt and osmotic stress

Salt and osmotic stresses cause ionic imbalances, leading to deformed chloroplasts, thylakoid swelling, and reduced grana stacks. These structural changes disrupt photosynthesis, limiting energy production. Both stresses also increase reactive oxygen species (ROS), causing oxidative damage to chloroplast components like lipids, proteins, and DNA. Stomatal closure under stress reduces CO_2_ availability, further impairing photosynthesis and increasing ROS production (Wang et al.). Chloroplasts activate antioxidant systems to neutralize ROS and maintain redox balance, and accumulate osmoprotectants like proline and glycine betaine to stabilize proteins and membranes (Wang et al.). These adaptive mechanisms are crucial for maintaining chloroplast function and plant survival under saline conditions.

## Nutrient availability

Plant nutrient deficiencies significantly impair photosynthesis by affecting energy production and growth processes. Nitrogen deficiency reduces, among others, chlorophyll synthesis, leading to chlorosis and decreased light absorption, while phosphorus deficiency hinders ATP formation, limiting energy availability (Therby-Vale et al., 2022). Magnesium is vital for chlorophyll structure and enzyme activation, so its absence diminishes photosynthetic efficiency (Meng et al.). Iron deficiency disrupts the electron transport chain, reducing energy transfer, and potassium plays a critical role in maintaining chloroplast structure, causing cell turgor loss and chloroplast damage (Therby-Vale et al., 2022). Additionally, micronutrients like zinc, manganese, and copper are crucial for enzyme function and overall plant health, and their lack further compromises photosynthesis (Therby-Vale et al., 2022). Addressing these deficiencies is essential for improving plant productivity and resilience.

## Atmospheric CO_2_ concentration

Carbon dioxide (CO_2_) concentration significantly affects chloroplast function and photosynthesis. Elevated CO_2_ levels increase substrate availability for Rubisco, reduce photorespiration, and improve the Calvin cycle’s efficiency. However, higher CO_2_ can also reduce stomatal conductance and limit CO_2_ diffusion if stomatal closure is excessive (Thompson et al., 2017). Long-term exposure to high CO_2_ also affects ROS balance, causing oxidative stress and may lead to down-regulation of photosynthetic capacity. Plants acclimate to elevated CO_2_ by adjusting photosynthetic machinery regulating gene expression and enzyme activities. Understanding these dynamics is essential for optimizing plant growth in the context of rising atmospheric CO_2_ levels (Xu et al., 2015).

## Conclusion

Chloroplasts, the crucial sites of many vital processes in plant cells, are highly sensitive to drastic changes in environmental conditions. Understanding the mechanisms underlying chloroplast differentiation and how different environmental factors influence chloroplasts is crucial for improving plant health and productivity, especially in changing environmental conditions. This collection of studies explores the various environmental factors affecting chloroplasts, the underlying mechanisms involved, and the adaptations chloroplasts use to cope with these conditions.

We would like to thank all authors for their contribution to this Research Topic and strongly believe that the presented results contribute to a better understanding of chloroplast functioning in the context of changing climatic conditions.
